# Lateral versus medial approach for total knee arthroplasty for valgus knee deformity shows comparable functional outcomes, hip–knee–ankle angle values, and complication rates: a meta-analysis of comparative studies

**DOI:** 10.1007/s00402-023-05088-2

**Published:** 2023-10-21

**Authors:** Michele Mercurio, Giorgio Gasparini, Olimpio Galasso, Filippo Familiari, Erminia Cofano, Valentina Sanzo, Gianluca Ciolli, Katia Corona, Simone Cerciello

**Affiliations:** 1 Department of Orthopedic and Trauma Surgery, “Magna Græcia” University, “Mater Domini” University Hospital, V.Le Europa, 88100 Catanzaro, Italy; 2 Department of Orthopaedics, A. Gemelli University Hospital Foundation IRCCS, Catholic University, Rome, Italy; 3 Department of Medicine and Health Sciences “Vincenzo Tiberio”, University of Molise, Campobasso, Italy; 4 Casa di Cura Villa Betania, Rome, Italy

**Keywords:** Total knee arthroplasty, Valgus knee deformity, Medial approach, Lateral approach, Knee society score, Hip–knee–ankle angle

## Abstract

**Introduction:**

The aim of this meta-analysis of comparative studies was to update the current evidence on functional and radiographic outcomes and complications between medial and lateral approaches for total knee arthroplasty (TKA) for valgus knee deformity.

**Materials and methods:**

The PubMed, MEDLINE, Scopus, and the Cochrane Central databases were used to search keywords and a total of ten studies were included. The methodological quality of the included studies was assessed. Data extracted for quantitative analysis included the Knee Society score (KSS), range of motion (ROM), surgical time, hip–knee–ankle angle (HKA), and number and types of complications. Random- and fixed-effect models were used for the meta-analysis of pooled mean differences (MDs) and odds ratios (ORs). The Mantel–Haenszel method was adopted.

**Results:**

A total of 1008 patients were identified, of whom 689 and 319 underwent TKA for valgus knee deformity with lateral and medial approach, respectively. The mean age was 70 ± 9.5 and 67.3 ± 9.6 years for the lateral and medial approaches, respectively. The mean follow-up was 37.8 ± 21.9 and 45.9 ± 26.7 months for the lateral and medial approach groups, respectively. Significantly higher functional outcomes were found for the medial approach, as measured by the postoperative KSS (MD = 1.8, 95% CI [0.48, 3.12], *P* = 0.007) and flexion ROM (MD = 3.12, 95% CI [0.45, 5.79], *P* = 0.02). However, both of these differences were lower than the minimal clinically important difference. Comparable surgical time and postoperative HKA angle values (MD = 0.22, 95% CI [− 0.30, 0.75], *P* = 0.40) between the two surgical approaches were found. The incidence of periprosthetic joint infections, fractures, transient peroneal nerve injuries, and deep vein thrombosis was comparable.

**Conclusion:**

This meta-analysis of comparative studies showed that when lateral and medial approaches are used for total knee arthroplasty for valgus knee deformity, comparable functional outcomes in terms of the KSS and ROM, surgical time, and postoperative hip–knee–ankle angle values can be expected. Similar rates of periprosthetic joint infection, fracture, and peroneal nerve injury were also found.

**Level of evidence:**

I.

**PROSPERO registration number ID:**

CRD42023392807.

**Supplementary Information:**

The online version contains supplementary material available at 10.1007/s00402-023-05088-2.

## Introduction

Valgus knees deformity accounts for 15% of all knees requiring a total knee arthroplasty (TKA) [1, 2]. TKA is an effective and safe procedure for managing patients with disabling articular pain, poor quality of life, and functional limitations arising from end-stage knee osteoarthritis (OA) [3], but one of the most controversial aspects for maximizing outcomes of valgus TKA depends on the surgical approach. Valgus knee deformity is characterized by a complex anatomy with a contracted ilio-tibial band, posterolateral capsule, posterior cruciate ligament, and lateral collateral ligament as well as osseous deficiency of the posterior lateral femoral condyle and medial collateral ligament laxity. Medial parapatellar arthrotomy has been the most commonly used approach and has been regarded as the gold standard for exposure of the knee joint [4]. Some authors have reported that the main risk associated with the medial approach for treating valgus knees deformity is an excessive release of medial structures including collateral [5]. Moreover, one study found that the anatomical axis was accurately restored in only 22–30% of valgus knees when a medial approach was used [6]. Restoration of the anatomical axis and correct ligament balance are important factors for stability and longevity of the prosthesis and for good functional outcome. The lateral parapatellar approach described by Keblish allows for a wider exposure of the lateral and posterolateral structures, which should be released for proper ligament balance [7]. Although the lateral approach is more technically demanding, the previous studies have reported promising results. Currently, the best surgical approach for valgus TKA is debatable, but new clinical comparative studies reporting outcomes of the medial and lateral approaches are emerging. However, most studies are negatively affected by small sample sizes and different methodologies which make it difficult to draw firm conclusions. The aim of this meta-analysis was to compare the functional and radiographic outcomes and complications between medial and lateral approaches for TKA in knees with valgus deformity. All available homogeneous data from comparative studies were pooled to identify differences between the two surgical approaches and to provide guidance to surgeons. The articles were selected based on the following PICO model: (P) patients with valgus knee deformity, (I) underwent TKA, (C) with the medial or lateral approach, and (O) assessed for functional and radiographic outcomes and complications.

## Materials and methods

### Search strategy

A systematic review of the published literature was conducted and reported according to the Preferred Reporting Items for Systematic Reviews and Meta-Analyses (PRISMA) statement. The PubMed, MEDLINE, Scopus, and Cochrane Central databases were searched in December 2022 with no lower date limit. The terms “total”, “knee”, “arthroplasty”, “replacement”, “valgus”, “lateral”, “medial”, “approach”, “outcome”, and “results” were used in different combinations to retrieve relevant articles. Two authors (M.M. and E.C.) independently conducted all the searches and screened the titles and abstracts to identify articles for inclusion. If a study could not be excluded based on the title and abstract, both reviewers reviewed the full text to reach a consensus on the inclusion or exclusion of the study, contacting a third senior author (O.G.) in case of major discrepancies. The reference list of each included article and the available gray literature at our institution were screened for the inclusion of potential additional articles.

### Inclusion criteria and study selection

The inclusion criteria were applied during the title, abstract, and full-text screenings; these were defined as follows: (1) observational studies [including case–control, cohort studies, and randomized-controlled trials (RCTs)]; (2) reporting of comparative outcomes and/or complications of medial versus lateral approaches for TKA in knees with valgus deformity; (3) reporting of > 10 surgically treated patients; and (4) articles written in English. The exclusion criteria were as follows: (1) unicompartmental knee arthroplasty (UKA) and (2) conversion from UKA to TKA. Other reviews, case reports, cadaveric or biomechanical studies, technical notes, editorials, letters to the editor, and expert opinions were excluded from the analysis but considered for the discussion section.

### Data extraction and quality assessment

Two authors (E.C. and M.M.) performed comprehensive data extraction from the included articles. The first author, journal name, year of publication, study design, patient demographics, type of surgical approach, type of implant, follow-up period, and complications were recorded. Data extracted for quantitative analysis included the Knee Society score (KSS), range of motion (ROM), the surgical time, hip–knee–ankle angle (HKA), and number and types of complications.

The methodological quality of the included studies was evaluated independently by three authors (E.C., M.M., and V.S.) with the modified Newcastle–Ottawa Quality Assessment Scale that classifies studies as “low” (0–3), “moderate” (4–6), and “high” (7–9) quality, based on the calculated score (Supplementary Table 1). The Cochrane risk-of-bias tool was used to evaluate the risk of bias in the included randomized-controlled studies [8] (Supplementary Tables 2 and 3). A substantial interobserver agreement (Cohen kappa coefficients ranging between 0.55 and 0.70) was reported.

### Data synthesis

All data were reported with one-decimal accuracy. The mean, standard deviation, and range were noted for the continuous variables and counts for the categorical variables. Functional and radiographic outcomes and complications were entered into a meta-analysis of pooled mean differences (MDs) and odds ratios (ORs), respectively. The Mantel–Haenszel method was adopted according to the Cochrane Statistical Methods Group. Random- or fixed-effect models were employed based on the between-trials heterogeneity as calculated by the *I*^2^ statistics; in particular, random-effect models were used when considerable heterogeneity was noted, unless the between-studies variance (*σ*^2^) was poor, in which case fixed-effect models were used despite the heterogeneity found. Review Manager (RevMan 5.3, Cochrane Collaboration, Nordic Cochrane Center, Copenhagen, Denmark) was used for statistical calculations; a *P* value < 0.05 was considered significant.

## Results

**Fig. 1:**
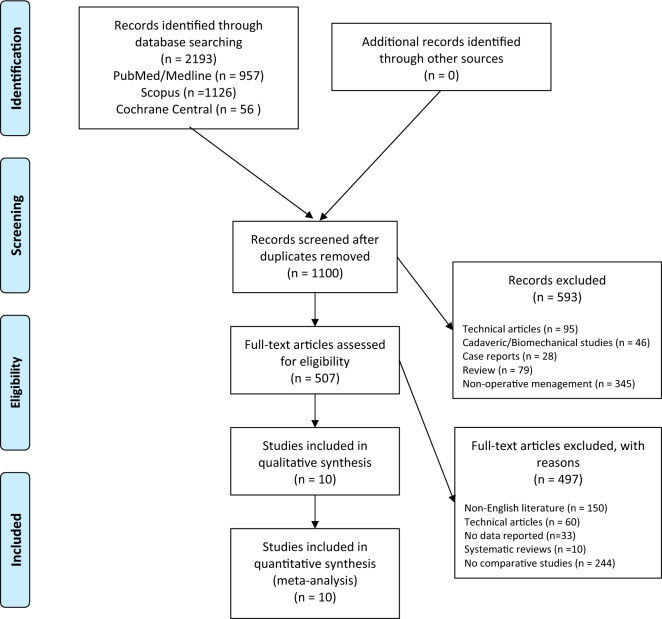
Preferred Reporting Items for Systematic Review and Meta-Analysis (PRISMA) flowchart for the search and identification of included studies. Source: Moher et al. For more information, visit http://www.prisma-statement.org

A total of 2193 relevant articles were identified through the initial search, 1100 abstracts were screened, and 507 full-text articles were assessed for eligibility based on our inclusion criteria, resulting in 10 comparative studies that were eligible for the meta-analysis (Fig. 1). The included studies were published from 2011 to 2022; 4 were randomized-controlled trials, and 6 were cohort studies, of which 2 were prospective and 4 were retrospective. Three studies were conducted in Europe, 3 in Asia, 2 in USA, 1 in Russia, and 1 in Brazil. A total of 1008 patients were identified, of whom 689 and 319 underwent TKA for valgus knee deformity with lateral and medial approaches, respectively. The most commonly reported diagnosis was primary osteoarthritis. Overall, female patients represented 298 (43.2%) and 182 (57.1%) of the lateral and medial approach cases, respectively. The mean age was 70 ± 9.5 and 67.3 ± 9.6 years for the lateral and medial approaches, respectively. The mean BMI was 27.9 ± 4.7 and 26.7 ± 4.7 for the lateral and medial approach groups, respectively. The mean follow-up was 37.8 ± 21.9 and 45.9 ± 26.7 months for the lateral and medial approach groups, respectively (Table 1).

**Table 1:** Characteristics of included studies

Author	Journal	Year of publication	Country	Approach	Patient demographics												Side %	
					Cases available (*N*)	Sex (%)		Age (years)			BMI			FU (months)				
						*M*	*F*	Mean	SD	Range	Mean	SD	Range	Mean	SD	Range	Left	Right
Dudek et al.	Journal of Clinical Medicine	2022	Poland	Lateral	143	141	2	68.2	11.2	NA	28.5	5.1	NA	60	24	24–120	NA	NA
				Medial	50	49	1	67.2	9.4	NA	28	4.2	NA				NA	NA
Filho et al.	Revista Brasilera de Ortopedia	2016	Brazil	Lateral	10	1	9	62.9	9.1	NA	29	3.9	NA	12	NA	NA	6	4
				Medial	11	1	10	62.6	10.6	NA	30.1	3.9	NA	12	NA	NA	3	8
Greenberg et al.	The Journal of Arthroplasty	2020	USA	Lateral	79	22	57	73	8	53–88	29.3	5.3	NA	22	18.9	NA	NA	NA
				Medial	25	6	19	65	11	40–80	NA	NA	NA	NA	NA	NA	NA	NA
Gunst et al.	International Orthopaedics	2015	USA	Lateral	315	60	255	70.9	9.4	NA	27.6	4.3	NA	34	3.4	NA	NA	NA
				Medial	109	24	85	68.1	11.2	NA	26.4	5.2	NA	61	4.2	NA	NA	NA
Guo et al.	International Journal of Surgery	2018	China	Lateral	13	1	12	68	NA	NA	NA	NA	NA	10	1.5	6–12	NA	NA
				Medial	18	1	17	65	NA	NA	NA	NA	NA	10	1.5	6–12	NA	NA
Kornilov et al.	Acta Orthopaedica	2015	Russia	Lateral	25	NA	NA	62	10	46–78	28	4.1	19–39	23	5	NA	NA	NA
				Medial	17	NA	NA	63.5	12	33–76	29	2.7	22–33	23	5	NA	NA	NA
Niki et al.	Knee Surgery Sports Traumatology Arthroscopy	2011	Japan	Lateral	26	5	21	68.9	4	39–84	23.9	3.6	17.1–31.6	3	NA	NA	NA	NA
				Medial	26	5	21	67.2	8.3	48–81	24	4.3	17.1–34.4	3	NA	NA	NA	NA
Nikolopoulos et al.	Knee Surgery Sports Traumatology Arthroscopy	2011	Greece	Lateral	22	9	13	76.5	5.5	59–81	NA	NA	NA	84	NA	NA	9	13
				Medial	22	7	15	73	5.8	57–80							12	10
Rawal et al.	The Open Orthopaedics Journal	2015	UK	Lateral	32	8	29	73.7	NA	NA	31.7	NA	NA	NA	NA	NA	NA	NA
				Medial	17	3	14	70.1	NA	NA	29.9	NA	NA	NA	NA	NA	NA	NA
Sekiya et al.	European Journal Orthopedics Surgery Traumatology	2014	Japan	Lateral	24	NA	NA	63	6.6	NA	23	NA	43.3	14.2	11.3	18–63	NA	NA
				Medial	24	NA	NA	66	6.7	NA	22.6	NA	43.2	8.4	11.3	18–63	NA	NA

*FU* follow-up, *SD* standard deviation, *BMI* body mass index, *NA* not available

Different types of implants were used for TKA, including the NexGen LPS (Zimmer, Warsaw, IN, USA) [9, 10], the posterior-stabilized tri-compartmental TKA (Tornier, Saint Ismier, France) [11], the posterior-stabilized prosthesis LPS-FLEX (Zimmer, Warsaw, IN, USA) [12], the Scorpio NRG^®^ posterior-stabilized prosthesis (Stryker Howmedica Osteonics, Allendale, NJ, USA) [13], the PCL retaining implant Foundation (Encore Medical, Texas, USA) [14], the PCL retaining implant Vanguard (Biomet INC., Warsaw, IN, USA) [14], the legacy constrained condylar knee-LCCK (Zimmer, Warsaw, IN, USA) [9], the NexGen-LCCK (Zimmer Biomet, Warsaw, IN, USA) [10], the rotating hinge knee-RHK implant (Zimmer, Warsaw, IN, USA) [9], the Modular III (MDT, Rio Claro, São Paulo, Brazil) [15], the cruciate-retaining CR types such as Sigma (DePuy Johnson & Johnson, Warsaw, IN, USA), NexGen CR (Zimmer Biomet, Warsaw, IN, USA), the AGC (Zimmer Biomet, Warsaw, IN, USA), and the LCS (DePuy Johnson & Johnson, Warsaw, IN, USA) [10].

### Functional outcomes

**Fig. 2:**
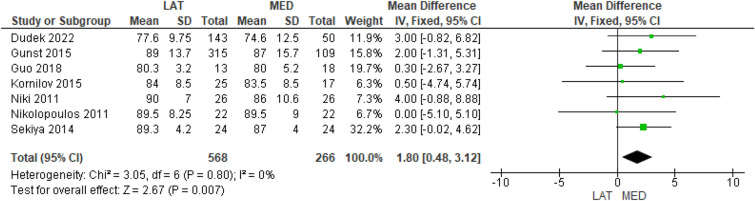
Comparison of the postoperative Knee Society Score between the lateral and medial approaches for total knee arthroplasty: forest plot of effect sizes

The preoperative KSS was reported in 8 studies [10–17] in 610 and 294 cases in the lateral and medial approach groups, respectively, and no difference was found between the groups (MD = − 1.20, 95% CI [− 2.91, 0.52], *P* = 0.17) (Supplementary Table 4); 7 studies reported the postoperative KSS [10–14, 16, 17] in 568 and 266 cases in the lateral and medial approach groups, respectively, and a statistically significant difference was found in favor of the medial approach (MD = 1.8, 95% CI [0.48, 3.12], *P* = 0.007) (Fig. 2).

**Fig. 3:**
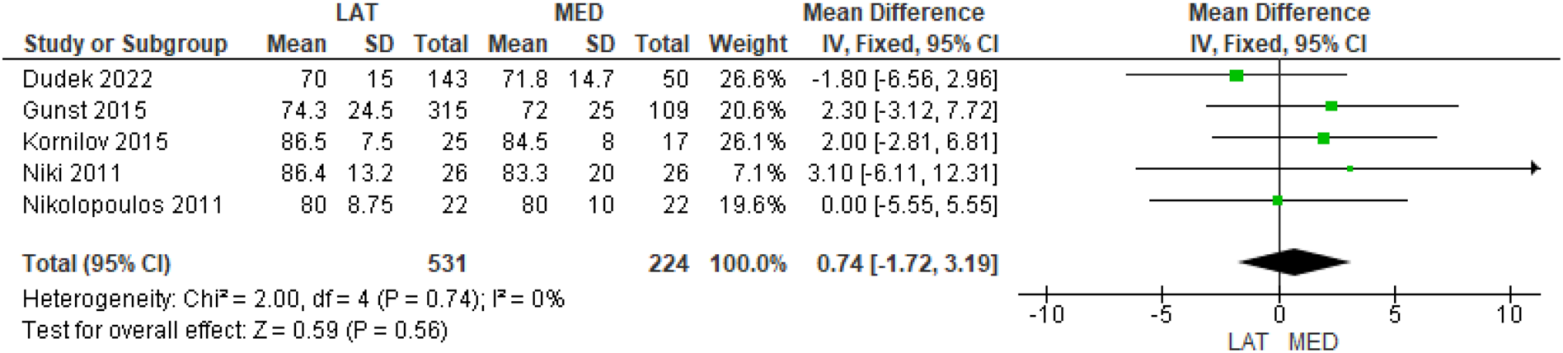
Comparison of the postoperative Knee Society Functional Score between the lateral and medial approaches for total knee arthroplasty: forest plot of effect sizes

Six studies investigated the preoperative KSS function score [10–12, 14–16] in 541 and 235 cases in the lateral and medial approach groups, respectively, and no significant difference was found between the groups (MD = − 1.56 95% CI [− 4.00, 0.88], *P* = 0.21) (Supplementary Table 5). Five studies investigated the postoperative KSS function score [10–12, 14, 16] in 531 and 224 cases in the lateral and medial approach groups, respectively, and no significant difference emerged (MD = − 0.74, 95% CI [− 1.72, 3.19], *P* = 0.56) (Fig. 3).

**Fig. 4:**
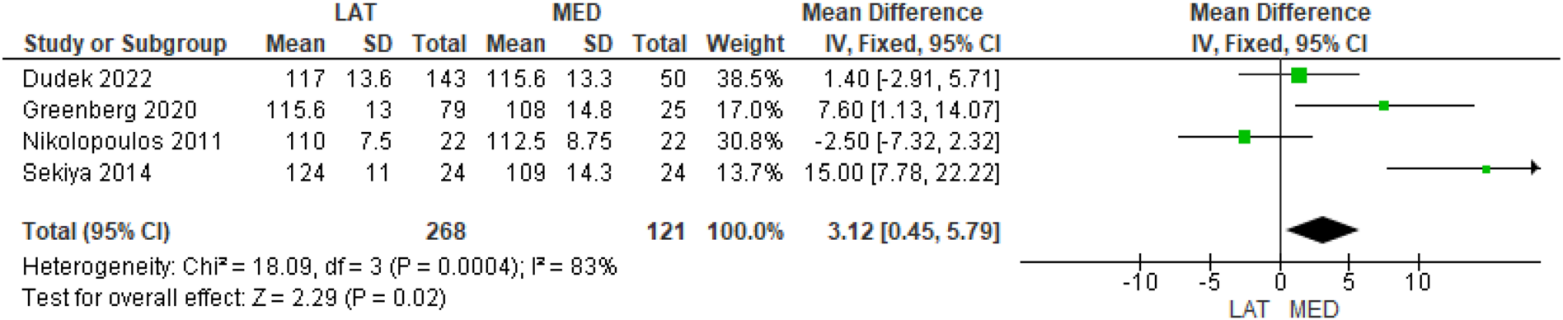
Comparison of the postoperative flexion range of motion between the lateral and medial approaches for total knee arthroplasty: forest plot of effect sizes

Five studies investigated the preoperative flexion ROM [9, 10, 13, 14, 16] in 293 and 138 cases in the lateral and medial approach groups, respectively, and four studies investigated the postoperative flexion ROM [9, 13, 14, 16] in 268 and 121 cases in the lateral and medial approach groups, respectively. No difference was found in the preoperative ROM values (MD = 0.82, 95% CI [− 1.77, 3.40], *P* = 0.54) (Supplementary Table 6) whereas a statistically significant difference in the postoperative ROM values was found in favor of the medial approach (MD = 3.12, 95% CI [0.45, 5.79], *P* = 0.02) (Fig. 4).

**Fig. 5:**
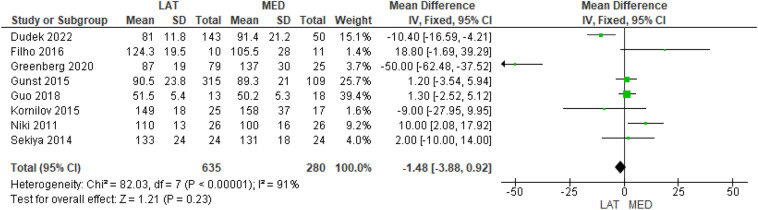
Comparison of the surgical time between the lateral and medial approaches for total knee arthroplasty: forest plot of effect sizes

**Fig. 6:**
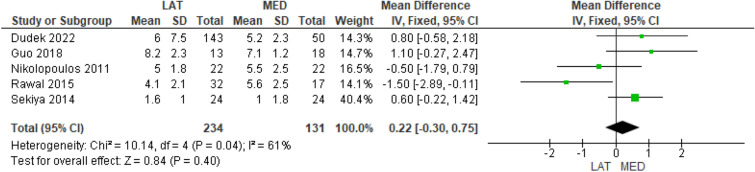
Comparison of the postoperative hip–knee–ankle angle between the lateral and medial approaches for total knee arthroplasty: forest plot of effect sizes

Eight studies investigated the surgical time [9–13, 15–17] in 635 and 280 cases in the lateral and medial approach groups, respectively, and no significant difference was found between the groups (MD = − 1.48, 95% CI [− 3.88, 0.92], *P* = 0.23) (Fig. 5). Finally, five studies investigated the pre- and postoperative valgus knee deformity by the HKA in 234 and 131 cases in the lateral and medial approach groups, respectively [13, 14, 16–18]; in the medial approach group, there was a higher preoperative valgus knee deformity (MD = 2.39, 95% CI [1.09, 3.69], *P* = 0.0003) (Supplementary Table 7), but no difference was found in the postoperative HKA values between the groups (MD = − 0.22, 95% CI [− 0.30, 0.75], *P* = 0.40) (Fig. 6).

### Complications

**Fig. 7:**
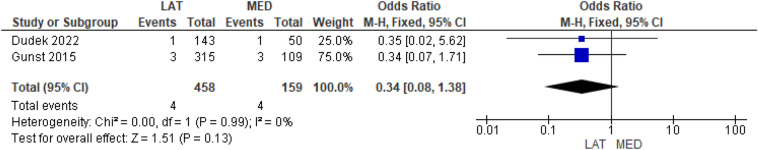
Comparison of the periprosthetic joint infection rate between the lateral and medial approaches for total knee arthroplasty: forest plot of effect sizes

The analysis showed no difference between the two groups in terms of the periprosthetic joint infection rate (OR = 0.34, 95% CI [0.08, 1.38], *P* = 0.13) (Fig. 7), as reported in two studies [11, 16] with rates of 2.8% and 1.5% for the lateral and medial approach groups, respectively.

**Fig. 8:**
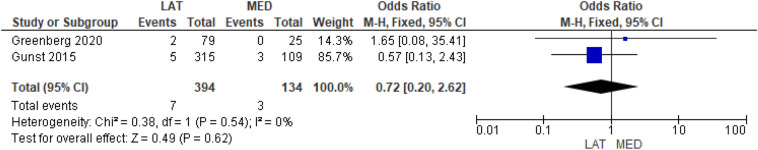
Comparison of the periprosthetic fracture rate between the lateral and medial approaches for total knee arthroplasty: forest plot of effect sizes

Periprosthetic fractures occurred in 1.8% and 2.2% of cases in the lateral and medial approach groups, respectively, as reported in two studies [9, 11], and no significant difference was found between the groups (OR = 0.72, 95% CI [0.20, 2.62], *P* = 0.62) (Fig. 8).

**Fig. 9:**
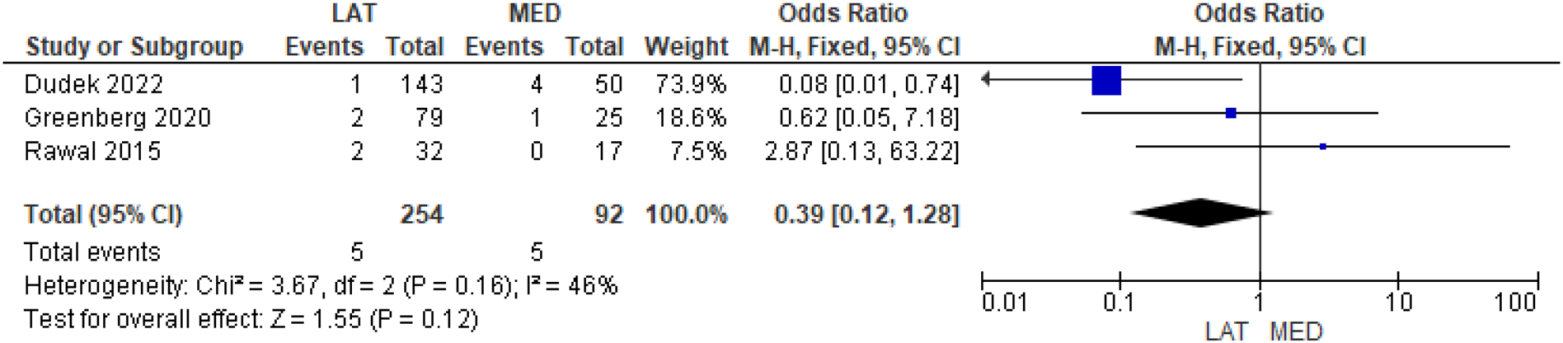
Comparison of the transient peroneal nerve injury rate between the lateral and medial approaches for total knee arthroplasty: forest plot of effect sizes

Three studies [9, 16, 18] reported a transient peroneal nerve injury with rates of 2% and 5.4% for lateral and medial approach groups, respectively, but no statistically significant difference between the two approaches was found (OR = 0.39, 95% CI [0.12, 1.28], *P* = 0.12) (Fig. 9).

Two studies [12, 14] investigated the occurrence of deep vein thrombosis, reporting rates of 12.5% and 14.6% for the lateral and medial approaches, respectively, and no significant difference was found between the groups (OR = 0.84, 95% CI [0.26, 2.70], *P* = 0.77) (Supplementary Table 8).

## Discussion

The most important finding of this study was that patients undergoing TKA with a medial approach exhibited, on average, a comparable postoperative KSS and flexion ROM to patients receiving TKA with a lateral approach. Similar surgical times, postoperative HKA angle values, KSS function scores, and complication rates were also observed for the two surgical approaches.

TKA for valgus knee deformity is a challenge. Technical aspects, such as surgical exposure, bone cuts, and ligament balancing, must be addressed to achieve the desired alignment and ligament balance. The choice of the surgical approach is a cornerstone in the context of preoperative planning. This study updates the current evidence on the medial versus lateral approach by including the largest number of comparative studies on the subject and analyzing for the first time the rate of specific complications. Two authors independently conducted all the searches; a comprehensive methodological quality assessment was also performed, and a substantial interobserver agreement was reported. While other systematic reviews [4, 19] have been reported on this topic, these have been mostly descriptive, including only some of the available data or aggregating data of heterogeneous noncomparative studies by applying techniques that do not align with conducting a meta-analysis, which creates a higher risk of bias.

A meta-analysis by Xu et al. [20] showed a better KSS and KSS function score in the lateral approach group; however, similar results for surgical time, ROM, correction of valgus knee deformity, and total complications were found for the lateral and medial approaches. A review by Wang et al. [19] suggested that the lateral approach combined with a tibial tubercle osteotomy or proximal quadriceps snip was more useful and safer than the medial approach in the treatment of severe uncorrectable valgus knee deformity in patients undergoing TKA. The present meta-analysis included a total of ten comparative studies, evaluating a higher number of clinical endpoints. In contrast to Xu et al. [20], we found higher postoperative KSS values in the medial approach group [13, 16, 17]. Notably, as reported by the Xu et al. [20], their meta-analysis was affected by a high degree of heterogeneity, having included the study by Langen et al. [21] who also considered knees with preoperative neutral alignment. Moreover, in the current meta-analysis, the difference (i.e., 1.7 points) between the groups was lower than the MCID, which was reported to be 9 [22]. Even the difference in flexion ROM was in favor of the medial approach, although it was small (i.e., 5°), raising the question of whether a significant *P* value may be too small for a patient to notice or consider relevant [23].

We also reported no differences in the surgical time, postoperative KSS function score or HKA angle values between the two surgical approaches. Eight studies investigated the surgical time [9–13, 15–17], and our results agree with those reported by the other studies [17, 20] refuting the hypothesis that a more challenging surgical exposure and soft-tissue closure increases surgical time in the case of a lateral approach. Notably, the Keblish approach is efficient, and the improvement in surgical instrumentation ensures satisfactory outcomes, especially when the surgeons are well trained [20].

A proper postoperative alignment is an important factor for the stability and longevity of the implant and for achieving satisfactory functional outcomes [4]. In the current meta-analysis, five studies investigated the pre- and postoperative valgus deformity by assessing the HKA. The medial approach group showed superior average preoperative valgus knee deformity, but the postoperative results were comparable between the two surgical approaches in terms of HKA values, indicating that the medial approach is reliable even in knees with valgus deformities. Furthermore, in addition to the surgical approach, many other factors should be considered when performing TKA for valgus knee deformity, such as component selection and degree of implant constraint, femoral anatomy, anterior–posterior axis for femoral component placement, and soft-tissue asymmetry [19]. Both cruciate-retaining and cruciate-sacrificing TKA implants were used in the studies included in the current analysis; however, it was not possible to assess the influence of these different implants on the postoperative HKA.

In the current meta-analysis, we found a comparable periprosthetic joint infection rate between the lateral and medial approach groups. Data on the infection rates were limited to only two studies; Gunst et al. [11] reported three (0.9%) periprosthetic knee infections in the lateral approach group and three (2.8%) cases in the medial approach group. The authors suggested that the equivalent surgical time and a perfect closure contributed to this result. Indeed, in the case of the Keblish approach, the arthrotomy can always be closed either by preserving the fat pad or performing a *Z*-plasty of the lateral retinaculum.

Next, we found comparable periprosthetic fracture, transient peroneal nerve injury, and deep vein thrombosis rates between lateral and medial approaches, which concurs with the results of other studies. Greenberg et al. [9] reported two intra-operative tibial fractures and one partial avulsion of the patellar tendon in the lateral approach group. The same authors also observed two cases of postoperative peroneal nerve palsy in the lateral approach group that recovered completely within 6 months of surgery, while one patient developed a foot drop in the medial approach group. Dudek et al. [16] showed one peroneal nerve palsy case with the lateral approach and four cases with the medial approach; in all these cases, the function of the nerve completely recovered. Therefore, peroneal nerve palsy is probably a consequence of the amount of intra-operative coronal correction rather than the result of the surgical approach.

Regarding the cases of deep vein thrombosis, the results we reported were mainly influenced by the data reported by Niki et al. [12] who noted five and seven cases of Doppler ultrasonography-confirmed deep vein thrombosis in the lateral and medial approaches, respectively; the authors classified these deep vein thrombosis as minor complications. Nikolopoulos et al. [14] reported only one deep vein thrombosis in the lateral approach group that was successfully treated.

The findings of this study should be interpreted concerning some limitations. First, we considered only English studies, potentially contributing to publication bias; moreover, although four recommended databases were used for the search, it is possible that further articles could have been found using other databases [24]. Second, a high degree of heterogeneity in terms of sample size between the included cohorts was found. Third, a different distribution between males and females was noted between the lateral and medial approach groups; therefore the results may not be generalizable. Fourth, the included articles contained a degree of heterogeneity in terms of the technique adopted for soft-tissue release of the lateral aspect of the knee and the type and degree of constraint of the implants. Finally, we included studies with different levels of evidence and evaluation times; it is likely that both functional outcomes and complications are affected by the length of patient follow-up, and these outcomes could also be potentially different between the two procedures if a specific follow-up time was determined. Nonetheless, major methodological strengths of this study are the comparative nature of the article inclusion strategy and the pooling of effect sizes to identify differences between the two surgical approaches.

## Conclusion

This meta-analysis of comparative studies showed that when lateral and medial approaches are used for valgus knees in total knee arthroplasty, comparable functional outcomes in terms of the KSS and ROM, surgical time, and postoperative hip–knee–ankle angle values can be expected. Similar rates of periprosthetic joint infection, fracture, and peroneal nerve injury were also found. Future studies including a larger number of patients undergoing total knee arthroplasty using the same implant and evaluated in long-term randomized-controlled trials should be conducted to confirm these findings.

### Supplementary Information

Below is the link to the electronic supplementary material.

Supplementary file1 (DOCX 112 kb)

## Data Availability

The data that support the findings of this study are included within the article and supplementary materials.
