# Supplementary material for: Lateral versus medial approach for total knee arthroplasty for valgus knee deformity shows comparable functional outcomes, hip–knee–ankle angle values, and complication rates: a meta-analysis of comparative studies
Source: Arch Orthop Trauma Surg. 2023 Oct 21;144(2):869–78. doi: 10.1007/s00402-023-05088-2 (PMC10822808; doi:10.1007/s00402-023-05088-2)
Supplement: Supplementary file 1 — Supplementary file1 (DOCX 112 kb) [file 402_2023_5088_MOESM1_ESM.docx]

**Lateral versus medial approach for total knee arthroplasty for valgus knee deformity shows comparable functional outcomes, hip-knee-ankle angle values, and complication rates: a meta-analysis of comparative studies**

Michele Mercurio ^1^, Giorgio Gasparini ^1^, Olimpio Galasso ^1*^, Erminia Cofano ^1^, Valentina Sanzo ^1^, Gianluca Ciolli ^2^, Katia Corona ^3^, and Simone Cerciello ^2,4^

^1^ Department of Orthopedic and Trauma Surgery, “Magna Græcia” University, “Mater Domini” University Hospital, V.le Europa, 88100, Catanzaro, Italy

^2^ Department of Orthopaedics, A. Gemelli University Hospital Foundation IRCCS, Catholic University, Rome, Italy

^3^ Department of Medicine and Health Sciences "Vincenzo Tiberio", University of Molise, Campobasso, Italy

^4^ Casa di Cura Villa Betania, Rome, Italy.

**Corresponding author**

- Olimpio Galasso
- Institutional address: Department of Orthopedic and Trauma Surgery, “Magna Græcia” University, “Mater Domini” University Hospital, V.le Europa, 88100, Catanzaro, Italy
- Phone: +39 0961 3647122
- Email: galasso@unicz.it

**E-mail address**

Michele Mercurio: [mercuriomi@gmail.com](mailto:mercuriomi@gmail.com)

Giorgio Gasparini: [gasparini@unicz.it](mailto:gasparini@unicz.it)

Olimpio Galasso: [galasso@unicz.it](mailto:galasso@unicz.it)

Erminia Cofano: [erminiacofano93@gmail.com](mailto:erminiacofano93@gmail.com)

Valentina Sanzo: va.sanzo@hotmail.com

Gianluca Ciolli: [gianluca.ciolli@gmail.com](mailto:gianluca.ciolli@gmail.com)

Katia Corona: [katia.corona@unimol.it](mailto:katia.corona@unimol.it)

Simone Cerciello: [simone.cerciello@me.com](mailto:simone.cerciello@me.com)

**1** Quality assessment of included studies according to the Modified Newcastle-Ottawa scale

| **Study Author (year)** | **Criteria** | | | | | | | | **Total** | **Quality** | |
| --- | --- | --- | --- | --- | --- | --- | --- | --- | --- | --- | --- |
|  | **1** | **2** | **3** | **4** | **5** | **6** | **7** | **8** |  |  |  |
| Dudek et al. (2022) | 1 | 0 | 1 | 1 | 2 | 1 | 1 | 1 | 8 | High |  |
| Greenberg et al. (2020) | 1 | 0 | 1 | 1 | 2 | 1 | 1 | 1 | 8 | High |  |
| Gunst et al. (2015) | 1 | 0 | 1 | 1 | 2 | 1 | 1 | 1 | 8 | High |  |
| Guo et al. (2018) | 1 | 0 | 1 | 1 | 2 | 1 | 1 | 1 | 8 | High |  |
| Niki et al. (2011) | 1 | 0 | 1 | 1 | 2 | 1 | 0 | 1 | 7 | High |  |
| Rawal et al. (2015) | 1 | 0 | 1 | 1 | 2 | 1 | 1 | 1 | 8 | High |  |

Based on the total score, quality was classified as “low” (0-3), “moderate” (4-6) and “high” (7-9).Criterion number (in bold): 1, representativeness of the exposed cohort; 2, selection of the non-exposed cohort; 3, ascertainment of exposure; 4, demonstration that outcome of interest was not present at start of study; 5, comparability of cohorts on the basis of the design or analysis; 6, assessment of outcome; 7, was follow-up long enough for outcomes to occur?; 8, adequacy of follow up of cohorts. Each study was awarded a maximum of one or two points for each numbered item within categories, based on the Modified Newcastle-Ottawa scale rules.

**2** Risk of bias graph: review authors' judgements about each risk of bias item presented as percentages across all included studies.


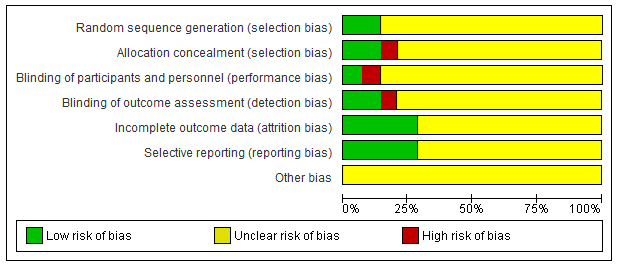


**3** Risk of bias summary: review authors' judgements about each risk of bias item for each included study.


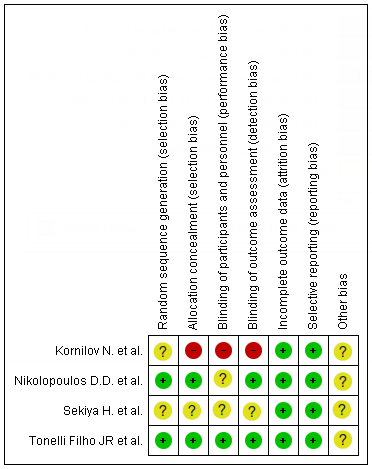


**4** Comparison of the preoperative Knee Society Score between lateral and medial approaches for total knee arthroplasty: forest plot of effect sizes.

CI means Confidence Interval


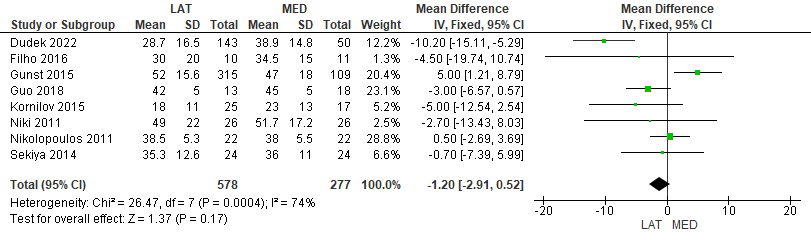


**5** Comparison of the preoperative Knee Society Functional Score between lateral and medial approaches for total knee arthroplasty: forest plot of effect sizes.

CI means Confidence Interval


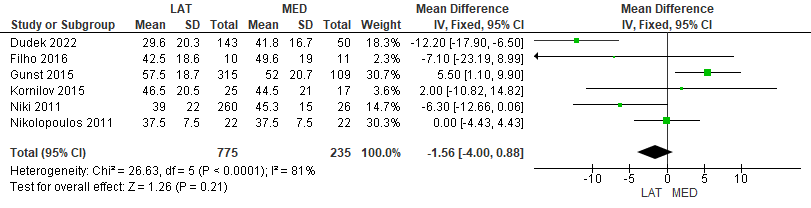


**6** Comparison of the preoperative flexion range of motion between lateral and medial approaches for total knee arthroplasty: forest plot of effect sizes.

CI means Confidence Interval


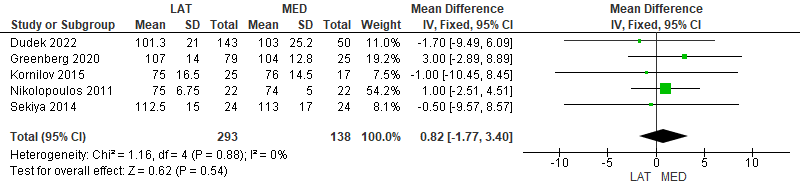


**7** Comparison of the preoperative hip-knee-ankle angle between lateral and medial approaches for total knee arthroplasty: forest plot of effect sizes.

CI means Confidence Interval


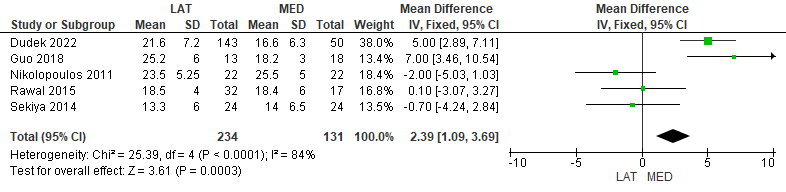


**8** Comparison of the deep vein thrombosis rate between lateral and medial approaches for total knee arthroplasty: forest plot of effect sizes.

CI means Confidence Interval.


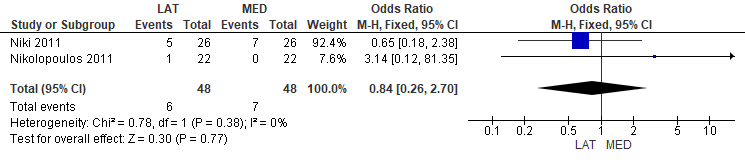


**10 GRADE**

**Checklist for the Quality Assessment Tool**

**Study limitations (Risk of Bias)**

1) Was random sequence generation used (i.e. no potential for selection bias)?

- Yes
- **Not for all**
- Unclear

2) Was allocation concealment used (i.e. no potential for selection bias)?

- Yes
- **Not for all**
- Unclear

3) Was there blinding of participants and personnel (i.e. no potential for performance bias)?

- Yes
- **Not for all**
- Unclear

4) Was there blinding of outcome assessment (i.e. no potential for detection bias)?

- Yes
- **Not for all**
- Unclear

5) Was an objective outcome used?

- **Yes**
- No

6) Were more than 80%^[[1]](#footnote-1)^ of participants enrolled in trials included in the analysis (i.e. no potential reporting bias)?

- **Yes**
- No
- Unclear

7) Were data reported consistently for the outcome of interest (i.e., no potential selective reporting)?

- **Yes**
- No
- Unclear

8) No other biases reported? (i.e. no potential of other bias)

- **Yes**
- No

9) Did the trials end as scheduled (i.e not stopped early)?

- **Yes**
- No

**Inconsistency^[[2]](#footnote-2)^**

1) Point estimates did not vary widely?

- **Yes**
- No

2) To what extent did confidence intervals overlap?

- Substantial overlap

(all confidence intervals overlap at least one of the included studies point estimate)

- **Some overlap**

(confidence intervals overlap but not all overlap at least one point estimate)

- No overlap

(At least one outlier: where the confidence interval of some of the studies do not overlap with those of most included studies)

3) Was the direction of effect consistent?

- **Yes**
- No

4) What was the magnitude of statistical heterogeneity (as measured by I^2^)?

- **Low (e.g. I^2^ <40%)**
- Moderate (e.g. I^2^ 40-60%)
- High (e.g. I^2^ >60%)

5) Was the test for heterogeneity statistically significant (p<0.1)?

- Not statistically significant
- **Statistically significant**

**Indirectness**

1) Were the populations in included studies applicable to the decision context?

- **Highly applicable**
- Applicable
- Poorly applicable

2) Were the interventions in the included studies applicable to the decision context?

- **Highly applicable**
- Applicable
- Poorly applicable

3) Was the included outcome not a surrogate outcome?

- **Yes**
- No

4) Was the outcome timeframe sufficient?

- **Sufficient**
- Insufficient

5) Were the conclusions based on direct comparisons?

- **Yes**
- No

**Imprecision**

1) Was the confidence interval for the pooled estimate not consistent with benefit and harm?

- Yes
- **No**

2) What is the magnitude of the median sample size?

- High (e.g. 300 participants)
- Intermediate (e.g. 100-300 participants)
- **Low (e.g. <100 participants)**

3) What was the magnitude of the number of included studies?

- Large (e.g. >10 studies)
- **Moderate (e.g. 5-10 studies)**
- Small (e.g. <5 studies)

4) Was the outcome a common event (e.g. occurs more than 1/100)?

- Yes
- No
- **Not applicable (i.e. not a dichotomous outcome)**

*Further optional question for those engaged in guideline development^[[3]](#footnote-3)^*

5) Was there no evidence of serious harm associated with treatment?

- **Yes**
- No

**Publication Bias (other considerations)**

1) Did the authors conduct a comprehensive search?

- **Yes**
- No

2) Did the authors search for grey literature?

- **Yes**
- No

3) Authors did not apply restrictions to study selection on the basis of language?

- Yes
- **No**

4) There was no industry influence on studies included in the review?

- **Yes**
- No

5) There was no evidence of funnel plot asymmetry?

- **Yes**
- No
- Unclear

6) There was no discrepancy in findings between published and unpublished trials?

- Yes
- No
- **Unclear**

1. 80% drop out is given as an example here a different proportion can be used depending on the context of the systematic review area [↑](#footnote-ref-1)
2. Reviewers may choose to use estimates from a subgroup analysis which may explain the inconsistency but should be cautious that such a explanation of heterogeneity may be due to the play of chance [↑](#footnote-ref-2)
3. This reflects GRADE guidance that guideline developers may use a less stringent threshold for judging imprecision of an intervention’s benefits when there is no evidence of harm compared with when judging the benefits of an intervention where there is strong evidence of harm [↑](#footnote-ref-3)
